# Illness-perception in adolescent attention-deficit/hyperactivity disorder: A qualitative study

**DOI:** 10.4102/sajpsychiatry.v29i0.2015

**Published:** 2023-03-28

**Authors:** Wanita Botha, Deborah van der Westhuizen

**Affiliations:** 1 Department of Psychiatry, Faculty of Health Sciences, University of Pretoria, Pretoria, South Africa

**Keywords:** ADHD, illness-perception, cognitive, behaviour, emotion, coping

## Abstract

**Background:**

Adolescents with attention-deficit/hyperactivity disorder (ADHD) experience aspects of their lives in diverse ways. They often have more energy and creativity which are positive traits of ADHD, while their inability to control their actions in academic or social spheres may cause feelings of inadequacy.

**Aim:**

To explore illness-perception, including emotional experiences, in adolescent ADHD.

**Setting:**

Using convenience sampling, 12 adolescent participants, eight boys and four girls, diagnosed with ADHD, were included. Participants followed up at Weskoppies Tertiary Psychiatric Hospital’s child-and-adolescent outpatient clinic.

**Methods:**

This qualitative study used semi-structured question guides to conduct interviews that allowed participants to actively construct their knowledge of their disorder. Maintaining a phenomenological perspective, thematical analysis of data was done.

**Results:**

Adolescents’ perceptions of their ADHD could be placed into three categories. ‘Negative self-perception in ADHD’ represented adolescents’ descriptions of ‘inability’ and ‘lack of control’ over their cognitive processes, behaviour, emotions, and restlessness. Adolescents perceived ‘feeling judged by others’, amplified self-stigmatisation and discrimination. This antagonistic environment caused conflict between their self-perception and others’ perception of them, further intensifying feelings of ‘not being normal’. The theme ‘self-empowerment strategies’, included controlling external stimuli, accepting support from others, and personalised learning strategies.

**Conclusion:**

Adolescents with ADHD struggle with cognitive, behavioural, and emotional control, and frequently experience stigmatisation and discrimination. They often learn to rely on self-taught coping strategies.

**Contribution:**

This research grants perspective to educators and clinicians on experiences of adolescents with ADHD, and identifies the need to address stigmatisation. It recognises the value of personalisation of coping methods.

## Introduction

Attention-deficit/hyperactivity disorder (ADHD) is one of the most prevalent neurodevelopmental disorders in childhood. Symptoms of inattention, hyperactivity, and impulsivity can present in a variety of ways.^1,2^ For diagnoses, symptoms should present in two settings, and result in impaired social and educational functioning.^2^ Benefit has been shown for early detection and implementation of preventative and non-pharmacological strategies.^1,3^ On the contrary, associations have been made between the risk of substance use disorders and undiagnosed and untreated ADHD.^4^ Behavioural development of children with ADHD often falls behind their expected developmental milestones, and repeated educational failures often worsen self-motivation. Certain symptoms of ADHD can present similarly to symptoms seen in other externalising disorders, wherein children may exhibit inappropriate behaviours towards their environment. These inappropriate behaviours include impulsivity, hostility, and irritability, and are frequently the parental reason for reaching out to mental health services.^5^ Adolescents with and without ADHD, go through similar processes of identity development. These processes include striving for independence, developing and establishing identity, forming work habits, planning for the future, and developing decision-making skills.^6^ The perception adolescents have of their ADHD is anticipated to influence how they perceive these developmental aspects and affect their functioning.^7^ Understanding their ADHD will help adolescents to develop an awareness of the effects it has on their lives, and to independently develop coping mechanisms. Illness-perception studies of ADHD in adolescents have largely been conducted in developed countries. Understanding the subjective experiences of adolescents who live with ADHD in South Africa, would help healthcare workers to understand and co-construct treatment modalities.

According to the Common-Sense Model (CSM) of illness perception and the associated illness-perception-questionnaire IPQ-R, people actively develop representations of their illnesses.^7,8^ These representations generally comprise of five key aspects, namely, identity, cause, timeline, consequences, and cure control.^9^ The illness-perception questionnaire has predominantly been used in medical illnesses but has become more popular in mental health research.^10^ In a systematic review, Wong et al. use the CSM to explore illness perceptions in children diagnosed with ADHD and their parents.^9^ Wong et al. further reflect on prior use of the CSM in Schizophrenia and Anorexia, and how there is benefit in including another key aspect of ‘emotional representations’ in assessments.^9^

Adolescents who accept their ADHD are more compliant with pharmacological and non-pharmacological interventions, yet, perceptions of illness may change over time, and some patients may become hopeless and give up.^9^ Discrepancies in perceptions of illness and achievements, may result in conflict between adolescents and parents.^11^ Perceptions of ADHD are likely to be influenced by external factors such as where children go to school and whether they have access to private mental health care. Limitations faced in South Africa’s public sector include a lack of resources like medication and specialised schools and programmes, in addition to a large, underdiagnosed population. Multidisciplinary involvement in the private sector is often limited, and may result in isolated pharmacological intervention.

In this illness-perception study, we qualitatively investigated illness-perception in adolescents with ADHD in South Africa. We asked adolescents how they perceive their symptoms and environment. We asked them whether they thought their emotions were affected by ADHD and what they found to be relieving factors and coping mechanisms.

## Research methods and design

This qualitative study was conducted from a constructivist worldview with a phenomenological approach. This approach forestalled to provide an in-depth understanding of the participants’ actual lived experiences as their environment presented itself.^12,13^ We conducted in-person semi-structured interviews to explore illness-perception and emotional representations in adolescents diagnosed with ADHD.

### Study setting

This study took place at Weskoppies tertiary psychiatric hospital, which receives referrals from the private and public health sectors. Weskoppies Hospital’s Child and Adolescent Clinic (WHCAC) provides care to approximately 250 children and adolescents monthly. Multidisciplinary psychiatric services are provided for various severities of mental illness, including, ADHD, mood-, psychotic-, anxiety-, behavioural-, and other neurodevelopmental disorders. Most of the patients have moderate or severe mental illness and are of both lower-and middle-class socioeconomic status. In this study, the participants’ severity of ADHD varied in complexity and outcome.

### Participants

We included adolescents who were 15 to 17 years old and diagnosed with ADHD by a psychiatrist and multi-disciplinary team using the Diagnostic and Statistical Manual of Mental Disorders, fourth edition (DSM-IV) or the Diagnostic and Statistical Manual of Mental Disorders, 5th edition (DSM-5) diagnostic criteria, and those with DSM-IV diagnostic criteria were subsequently confirmed with DSM-5 criteria.^2^ Participants had to be stabilised on treatment for ADHD and comorbidities, and compliant with follow-up. We excluded participants with comorbidities such as autism spectrum disorder, moderate to severe intellectual disability, and language disorders. Due to the frequency of comorbidity, mood disorders were not excluded. The typical episodic nature of mood disorders and the longstanding course of ADHD were considered, and both conditions needed to be stabilised on treatment. We also excluded patients presenting for first evaluations. Lastly, participants had to be able to converse in English or Afrikaans, as understandable to the researcher.

To limit selection bias, we used convenience sampling, and approached 17 parent-adolescent pairs, at WHCAC. Thirteen parent-adolescent pairs showed interest and agreed to participate. The study was verbally explained while referring to an information brochure as a visual aid. Provisions were made for questions or clarifications, and consent, assent, and withdrawal processes were discussed. To limit observation bias, participants were interviewed without their parents. Question-guided interviews, led by the principal researcher, were based on the IPQ-R, and where needed, a simplified version of the IPQ-R (Table 1). We excluded one audio recording due to technical difficulties. Data collection continued until data saturation, resulting in 12 participants being included in the final analysis.^13^

**TABLE 1:** Simplified interview framework for illness perception in adolescents with attention-deficit/hyperactivity disorder.

Key aspect categories	Simplified question
Illness perception	1. What is ADHD?
	2. Where does ADHD come from?
	3. How long does ADHD carry on?
	4. How is your life different because of ADHD?
	5. What makes it easier for you to live with ADHD?
Emotional representations	6. How are your emotions affected by ADHD?
Coherence	7. How well do you think you understand ADHD?

*Source:* Adapted from Moss-Morris R, Weinman J, Petrie K, Horne R, Cameron L, Buick D. The revised Illness Perception Questionnaire (IPQ-R). Psychol Health. 2002;17(1):1–16. https://doi.org/10.1080/08870440290001494 and Wong IY, Hawes DJ, Clarke S, Kohn MR, Dar-Nimrod I. Perceptions of ADHD among diagnosed children and their parents: A systematic review using the common-sense model of illness representations. Clin Child Fam Psychol Rev. 2018;21(1):57–93. https://doi.org/10.1007/s10567-017-0245-2

ADHD, attention-deficit/hyperactivity disorder.

### Interviews

During planning and peer debriefing, we recognised a need to simplify the CSM IPQ-R. The integrity of the concepts of identity, cause, timeline, consequences, cure control, emotional representations, and coherence, was maintained; however, some participants required questioning by using simplified terminology (Table 1).

The IPQ-R and interview question framework guided the principal researcher to assist participants in exploring perceptions and understanding of their ADHD, while remaining on topic, and expanding with follow-up questions. Where participants did not understand, the interviewer simplified or clarified questions. We enhanced participants’ comfort and openness by employing interview methods such as paraphrasing, clarification, and gesturing continuance. All the interviews were audio-recorded and the interviewer made field notes, to track her thoughts, and note participants’ non-verbal cues. Peer debriefing resulted in questions being added to the interview framework as more codes arose and clarifications were required.

Data were collected from November 2020 to March 2021. Audio recordings lasted from 13 min to 36 min, with a median duration of 26 min. Verbatim transcriptions and required translations were done by W.B., with line numbers assigned to questions and participant responses. Each participant was assigned a pseudonym, correlating with their gender and ethnicity, to provide a ‘real-life’ experience for the reader.

### Data analysis

Data analysis and management comprised of typical thematical analysis.^14^ Initial data revisions drew on manual exploration, and ATLAS.ti software was used for further full scale coding and clarifications. Data familiarisation comprised of revising the raw data while simultaneously listening to recordings, reading verbatim transcriptions, and reviewing field notes. ATLAS.ti was used to review participants’ first-person, subjective perceptions of the different aspects of ADHD. With open coding of transcribed data, concepts that expressed similar feelings, experiences, and ideas, were highlighted and organised as a condensed whole. Axial coding was used to group codes into themes and sub-themes, with comparisons between interviews. Irrelevant or unclear themes were discarded. Themes were organised by analysing and identifying re-emerging patterns, then either merging, separating, or improving themes across participants’ transcriptions. Themes and sub-themes were refined to ensure a precise understanding of the message relayed by the data and to ensure accurate expression of the participants’ experiences of specifically their ADHD. Concise and logical naming of themes was done, and an audit trail was kept. The Standards for Reporting Qualitative Research guided the reporting of results.^15^

### Ethical considerations

Approval for this research was obtained by the Faculty of Health Sciences MMed Committee and from the University of Pretoria Ethics Committee (Ethics Reference No: 652/2020).

As minors with mental illness represent a vulnerable population, written consent and assent were obtained from parents and adolescent participants after explaining the study. Participants were assigned an alphanumerical code to ensure anonymity. Safeguarding precautions were implemented to prevent unjustifiable risk or harm to participants, and secure storage of data was done according to the university’s policies.

## Results

Seven of the 12 participants had comorbidities, mainly comprising of mood disorders (Table 2).

**TABLE 2:** Demographics, comorbidities and age at diagnoses of adolescents with attention-deficit/hyperactivity disorder who participated in this qualitative study.

Participant number	Pseudonym	Gender	Age	Diagnoses	Age at diagnoses
P1	Mia	Female	15 years	ADHD, UBD	6 years
P2	Junior	Male	17 years	ADHD, EP, MDD	6 years
P3	Olivia	Female	17 years	ADHD, PDD	13 years
P4	Andre	Male	16 years	ADHD, UBD	12 years
P5	Gerhard	Male	15 years	ADHD, UBD, CD	5 years
P6	Christo	Male	17 years	ADHD	6 years
P7	Sibongile	Female	15 years	ADHD	12 years
P8	Jerome	Male	15 years	ADHD, UDD	9 years
P9	Liam	Male	15 years	ADHD	12 years
P10	Bandile	Male	16 years	ADHD	13 years
P11	Eugene	Male	15 years	ADHD	13 years
P12	Heidi	Female	17 years	ADHD, UBD	6 years

ADHD, attention-deficit/hyperactivity disorder; UBD, unspecified bipolar and related disorders; EP, epilepsy; MDD, major depressive disorder; PDD, persistent depressive disorder; CD, conduct disorder; UDD, unspecified depressive disorder.

Field notes reflected that even though the participants denied feeling restless, they fidgeted with their hands, rocked on chairs, or tapped their feet. Participants struggled to maintain attention with long explanations and often came across as childlike.

Three themes with subthemes emerged from patient interviews. The three themes included ‘negative self-perception in ADHD’, ‘feeling judged by others’ and ‘self-empowerment strategies’.

### Theme 1: Negative self-perception in attention-deficit/hyperactivity disorder

We identified four subthemes under this theme: (1) lack of control of cognitive processes, (2) lack of behavioural control, (3) emotional dysregulation, and (4) somatic discomfort with activity restriction.

#### Lack of control of cognitive processes

Participants described a discrepancy between the control they desired to have over their cognitive processes and actions, in contrast to their perceived focus and expressed behaviour. They described cognitive processes as ‘their brain switching-off’, ‘thoughts jump’, or ‘zoning-out’:

‘In class, I would concentrate for 5 or 10 minutes, and then my brain switches off and I start to think about other things. Then it takes me a while to concentrate on schoolwork again.’ (P4, Andre, male)

‘Uhm, my thoughts jump around, and I can think of the most random things… I don’t know, and I will go too deep into conversations, or, and when someone will be talking to me about something I will zone out and not even know what they are saying, and I’ll just agree to everything… I won’t really… I’ll hear what is happening, but I won’t really listen to it.’ (P3, Olivia, female)

External stimuli frequently resulted in unwanted distractions:

‘And then when the answer is in my head and then the teacher talks to me, then it will just disappear.’ (P5, Gerhard, male)

‘… [*I*]f you are distractible, and you can’t concentrate in class, and you can’t sit still, something pulls your attention away.’ (P11, Eugene, male)

Poor concentration, daydreaming, and a reduced work pace, caused participants to fall behind:

‘I think that people get a lot further than me… so I can still be stuck on certain things for a very-very-very long period of time. So, I feel like I then fall behind, and then it becomes more panic… so, I would have to work a lot harder.’ (P3, Olivia, female)

‘I wish they knew, how long it actually takes people with ADHD to grasp certain things. So, if someone is speaking about physics or something, it takes me a lot longer, than it takes my sister, for instance, normal people. So, I wish people understood, I’m not stupid, it’s just that, I don’t take, I’m not as fast as you guys are.’ (P3, Olivia, female)

#### Lack of behavioural control

Participants described how their uncontrolled behaviour was often perceived as naughty, spiteful, or on purpose:

‘Uhm, a lot of the time, people think that I am trying to be naughty, but also, I don’t really have great control over certain things…’ (P3, Olivia, female)

‘It’s weird because I want to stand, but my mind is telling me to sit down but I want to stand so I’m going to stand. I’m disobeying my own mind.’ (P10, Bandile, male)

#### Emotional dysregulation

The most prominent emotional expressions were anger and irritability:

‘Just a severe irritation or aggression. One of those two. Because, I am trying to concentrate, and then now, maybe I just got to concentrating, and now all of a sudden, the dog is barking.’ (P12, Heidi, female)

‘… But, because I don’t understand things as fast, I get more irritated, then I feel like it relates to my mood at the same time.’ (P3, Olivia, female)

One participant continued to describe the unpredictability of emotional dysregulation as a ‘roller-coaster’ (P1, Mia, female).

#### Somatic discomfort with activity restriction

Participants reported physiological discomfort and a feeling of being trapped, when fiddling was restricted or they were told to ‘sit still’:

‘So, when I am forced to stay still… it gives a build-up in my stomach, and it almost makes me feel like screaming… I get more aggressive if I get forced to stay still because then I’ve got no, there’s no positive way to take it out… The stress inside my body.’ (P3, Olivia, female)

‘Then it feels… when the teacher said I am not allowed to go out, it felt like I almost got a heart attack…’ (P6, Christo, male)

### Theme 2: Feeling judged by others

Two subthemes emerged from this theme: (1) self-stigmatisation of ‘not normal’ and (2) discrimination.

#### Self-stigmatisation of ‘not normal’

Participants were soberly aware of their differences compared to peers and often felt stigmatised, misunderstood, or *not normal*:

‘Doesn’t mean when you have ADHD, you have to be treated differently because of the way you are. It’s just something that needs somebody to understand in order to help that person. Doesn’t mean that when you have this type of ADHD, you’re not human…’ (P7, Sibongile, female)

‘Because I struggled to concentrate in class, I felt dumber than the others.’ (P12, Heidi, female)

#### Discrimination

Participants’ behaviour exaggerated their teachers’ and peers’ frustration; this resulted in the participants experiencing the classroom as even more challenging:

‘Well, a lot of people get irritated with me, so a lot of people will be like “I can’t deal with her”. So, then they’ll just leave me. So, a lot of people leave because of it.’ (P3, Olivia, female)

‘The teacher thinks I’m disrespectful and he thinks that I think I know better than him, and it’s not that. I tried connecting with a teacher this year, but he took it out straight in front of the whole class. He’s like “You have mental problems”.’ (P10, Bandile, male)

‘I see the classroom as a football pitch. You know the opponent is coming for you so you should think who to pass the ball to, and if you should run with the ball, go and score, you see?’ (P10, Bandile, male)

### Theme 3: Self-empowerment strategies

Three subthemes emerged from this theme: (1) controlling external stimuli, (2) accepting support from others, and (3) personalised learning strategies.

#### Controlling external stimuli

Participants described the advantage of external stimuli on concentration. Stimuli included movement or fidgeting, eating, and music:

‘Besides the pills, toys, fidget toys are very helpful… and bubble-wrap, even though it does make very weird noises or ice. So, when you put ice into a glass, and it slowly starts melting, I can hear it. I can hear, the, it fall onto each other. So that helps, then I just concentrate on that.’ (P3, Olivia, female)

Two participants described how exercise improved emotional dysregulation and relieved restlessness:

‘Concentration, not a lot. But aggression decreases. It, helps me calm down. Yes, it stretches through the whole day that I stay calm after I have exercised.’ (P12, Heidi, female)

‘I play violent sport, so I play, water-polo, national water-polo, so when I started water-polo, it helps a lot more, because I’m taking it out healthy, like I am taking out whatever energy I had, in a healthier way than I used to.’ (P3, Olivia, female)

Focused improved for two participants who had snacks, or had their hunger satiated:

‘Mostly in class, I always have a packet of sweets and snacks. I know teachers don’t allow us to eat in class, but I eat anyway even though I know that they don’t allow me. The teacher won’t even recognize me because he’s used to me being loud now, all of a sudden, I’m quiet because I’m keeping myself busy with the food. Yes. It helps me sit still and I do concentrate because when I’m keeping still, I don’t talk to anyone, I listen.’ (P10, Bandile, male)

‘If I don’t eat, then I really can’t concentrate because my stomach burns. Like, stomach cramps, stomach burns if I don’t eat.’ (P5, Gerhard, male)

A constant background stimulus, for instance, listening to music on headphones, often drowned out distractions:

‘If I listen to music … Especially in class as well, then I listen first, then when they say we can do our work, then I am allowed to use my headphones, and then it is more-easier for me, as to hearing the class that is going to distract me, to having to talk with them.’ (P9, Liam, male)

‘But the strange thing to me is, if I have in headphones, then I can concentrate. Then I can’t hear other noises around me, except the music in my ears … Because my MP3 player broke, so I did long ago. And that is when it snapped, that it actually helps me more.’ (P12, Heidi, female)

#### Accepting support from others

The presence of parents or teachers during schoolwork motivated multiple participants and reminded them to maintain focus.

‘My mom, one of my teachers, well, often, if she can see me drift off, she would hit my table, not like, not violently, but she would be like, you know. Uhm, but, other than that, no, not really.’ (P3, Olivia, female)

‘Oh, my God. You’re going to end up doing things out of this world. Because having support while having ADHD, it can help you get treatment, it can help you to get well, it can help you know what wrong you’re doing and what right thing you’re doing, but not getting in a supportive environment, you going to end up doing things that are out of the way.’ (P7, Sibongile, female)

Participants also stated how medication assisted them with their cognitive and emotional struggles.

‘And then it just helps, a lot more, with my concentrating on certain things.’ (P3, Olivia, female)

‘Like, it helps to keep me calmer or to concentrate or something like that.’ (P5, Gerhard, male)

‘When I drink the pills, it helps me to be relaxed, but if I don’t drink the pills, I am out of my box.’ (P11, Eugene, male)

‘It helps me not to become aggressive so easily.’ (P4, Andre, male)

‘If I drink my pills, I am happy always.’ (P11, Eugene, male)

#### Personalised learning strategies

Participants explained that practical and interest-based learning enhances education. One participant said that he enjoyed welding, as it ‘keeps his hands busy’ (P5, Gerhard, male). Another participant flourished in an art-based school (P7, Sibongile, female). Home-schooling provided an alternative platform for two participants to formulate structure and boundaries. Learning was further enhanced by practical methods such as colour-coding, breaking homework into smaller steps, drawing, or using pictures. An organised environment also enhanced participants’ learning.

‘… [*T*]eachers helped me because I have ADHD and struggle to concentrate… they gave me only a little bit of work to study every day and then the next day a little bit because if I had to study everything at once my brain would switch off and I would struggle to concentrate. The teachers decided they will break the work down into little pieces so I can do the work.’ (P4, Andre, male)

‘Anatomy, the one that I am doing now. It is easier to color in the muscle, and then to study it according to that color. So, coloring-in is one of them. And, like in my room, I pack certain things in certain places and it has to stay there.’ (P12, Heidi, female)

## Discussion

In this qualitative study, we explored the various ways in which adolescents with ADHD from Pretoria, South Africa perceived the impact of ADHD on their lives, how it affected their emotions, and what they experienced as coping strategies. The participants’ perceptions regarding their ADHD were explored in a setting where adolescents are required to self-adapt to a resource-deprived system. This system often struggles to accommodate children with different learning strategies or concentration levels.

Adolescents with ADHD’s feelings of loneliness often get amplified by not being in control of the cognitive, behavioural, and emotional aspects of their lives.^16^ In our study, adolescents felt that their environment expected them to sit still, be quiet and concentrate. Cognitively, concentration difficulties were expressed as attention being drawn away or ‘zoning out’, which often caused them to fall behind. Behaviourally, adolescents struggled to control their psychomotor activity, felt restless, and were unable to ignore external stimuli. The restricting activity of these adolescents resulted in intense physiological and mental discomfort. Adolescents with ADHD are known to experience feelings of irritability and aggression and have somatic complaints.^16,17^ We also found that emotional dysregulation correlated with previously described ‘unpredictable shifts’ to ‘unusually intense’ reactions, that were ‘disproportionate’ to developmental age and the situational trigger.^17,18^ Emotional dysregulation may make it harder for adolescents to recognise, describe or think about their emotions. In our study, some adolescents showed improved emotional regulation and less aggression on stimulant medication, specifically long-acting methylphenidate.^19,20^

Adolescents with ADHD often perceive themselves as ‘not normal’ and as in other studies, this often resulted in them feeling excluded, which leads some of them to conceal their diagnosis.^21,22,23^ These adolescents are generally aware of their shortcomings and are concerned about their futures.^22^ They compare themselves to their peers and find the classroom to be consistently challenging. Our findings corroborated with findings by Schoeman et al., where participants felt different, labelled, or misunderstood, and thus felt stigmatised and were treated differently by a discriminating environment.^24^ This misunderstanding has been expressed in previous research, where teachers often associated behaviour problems with an impaired family structure or the ‘problem child’.^25^ In the same study, learners with ADHD often misbehaved when they perceived the absence of valuable subject content, or experienced peer pressure, emotional turmoil, or stress caused by lessons.^25^ Teachers may not always understand ADHD and may use harsher discipline measures including name-calling, cursing, and degrading the upbringing of adolescents with ADHD.^25,26^ Mitzi et al. describe how adolescents with ADHD with improved self-awareness, had positive cognitive and social experiences.^27^ Adolescents with ADHD have to learn to control their symptoms, overcome struggles, build resilience, and develop coping strategies to enhance performance.^22^ In our study, adolescents with ADHD had to learn to cope with external environmental distractions. They found that controlled external stimuli such as listening to music via headphones would ameliorate multiple distractions such as teachers or peers talking or something passing by. Other beneficial external stimuli included eating and movement and practical methods like physical subjects, colour-coding, shapes, and breaking tasks into steps. Adolescents with ADHD typically lose motivation when tasks are lengthy and boring, but are motivated by variability in tasks.^28^ These adolescents are also motivated by adult support and positive reinforcement through social or monetary means.^28^ Our research correlated with the benefit of developing routines and problem-solving strategies in adolescents with ADHD.^18^ Adolescents with ADHD experience rules, regulations, routines, and an organised environment, conducive to both cognitive performance and emotional regulation. Adolescents who participated in the formulation of their routine felt an enhanced sense of responsibility and self-awareness. Parents and teachers who create an organised environment and establish a routine, rather than relying on punitive discipline, also have favourable outcomes for the education of adolescents with ADHD.^25^

Physical exercise is a known coping strategy for adolescents with ADHD; it improves impulsivity and behaviour, and in some cases improves attention, in both reading comprehension and mathematics.^29,30^ In our study, adolescents reported that exercise improved their mood, but not their concentration.

Although ADHD is often perceived to be an externalising illness, adolescents in this study communicated internal struggles. They perceived a lack of control over- and unpredictable cognitive processes, behaviour, and emotions. In this study, adolescents perceived their environment to be distracting and negatively expectant. These adolescents were exhausted by having to maintain attention and keep up with the rest of the class. Remaining still while feeling restless often led to physiological discomfort. These internal experiences may result in unpredictable and often misunderstood behaviour, which others then classify as externalising symptoms. As externalising symptoms manifest, adolescents with ADHD may be discriminated against. Adolescents without good support structures or coping mechanisms may find themselves in negative, symptom-provoking discrimination cycles which will amplify their feelings of abnormality. We recommend that caregivers should consider how the environment can distract a child with ADHD rather than labelling a child with ADHD as the distraction.^25^

### Strengths and limitations

This study explores the benefits of assessing illness-perception of adolescents with ADHD, in a South African context. It considers how the exploration of illness-perception might provide a platform for understanding and individualising adherence and coping strategies. This study corroborates previous global research on illness burden and discrimination and adds to the limited data available in the South African context. Several limitations are acknowledged in this study, including small sample size and short duration of some interviews; however, interviews continued until data saturation was reached. Generalisability is limited due to the participants in this study representing patients attending a single tertiary psychiatric hospital in the resource-limited public sector in South Africa, who are typically of lower socioeconomic status. However, overlapping experiences have been found in resource-rich developed countries.^31^ Yet, patients with private medical insurance may have different experiences of their ADHD. Due to the nature of qualitative research, the limitations to statistical validity and objectivity should be considered when reading this study. More than half of the adolescent participants in our study had comorbidities, most frequently mood disorders. Even though these comorbidities can be expected in ADHD, and both ADHD and comorbidities were required to be stabilised as inclusion criteria, this needs to be taken into consideration when interoperating the study.

## Conclusion

Our findings reveal that adolescents with ADHD in South Africa struggle to control their cognitive, behavioural, and emotional spheres. Our findings corroborate previous research which describes mood aspects, emotional dysregulation, stigmatisation, and discrimination – the latter enhancing the feelings of being ‘not normal’. Adolescents with ADHD can identify coping strategies and relieving factors, including controlling external stimuli, accepting support from others, and personalised learning strategies This qualitative study supports the premise that the external environment is distracting for adolescents with ADHD. Future research should focus on how the individualised illness-perceptions of ADHD, might assist healthcare workers and parents, to co-construct modalities to enhance adherence, coping, and address stigmatisation in resource-deprived settings.
